# Multi-scenario pear tree inflorescence detection based on improved YOLOv7 object detection algorithm

**DOI:** 10.3389/fpls.2023.1330141

**Published:** 2024-01-22

**Authors:** Zhen Zhang, Xiaohui Lei, Kai Huang, Yuanhao Sun, Jin Zeng, Tao Xyu, Quanchun Yuan, Yannan Qi, Andreas Herbst, Xiaolan Lyu

**Affiliations:** ^1^School of Agricultural Engineering, Jiangsu University, Zhenjiang, China; ^2^Institute of Agricultural Facilities and Equipment, Jiangsu Academy of Agricultural Sciences, Nanjing, China; ^3^Key Laboratory of Modern Horticultural Equipment, Ministry of Agriculture and Rural Affairs, Nanjing, China; ^4^Institute for Chemical Application Technology of JKI, Braunschweig, Germany

**Keywords:** pear tree inflorescence, long-distance detection, YOLOv7, EMA, SPPCSPCS, Soft-NMS

## Abstract

Efficient and precise thinning during the orchard blossom period is a crucial factor in enhancing both fruit yield and quality. The accurate recognition of inflorescence is the cornerstone of intelligent blossom equipment. To advance the process of intelligent blossom thinning, this paper addresses the issue of suboptimal performance of current inflorescence recognition algorithms in detecting dense inflorescence at a long distance. It introduces an inflorescence recognition algorithm, YOLOv7-E, based on the YOLOv7 neural network model. YOLOv7 incorporates an efficient multi-scale attention mechanism (EMA) to enable cross-channel feature interaction through parallel processing strategies, thereby maximizing the retention of pixel-level features and positional information on the feature maps. Additionally, the SPPCSPC module is optimized to preserve target area features as much as possible under different receptive fields, and the Soft-NMS algorithm is employed to reduce the likelihood of missing detections in overlapping regions. The model is trained on a diverse dataset collected from real-world field settings. Upon validation, the improved YOLOv7-E object detection algorithm achieves an average precision and recall of 91.4% and 89.8%, respectively, in inflorescence detection under various time periods, distances, and weather conditions. The detection time for a single image is 80.9 ms, and the model size is 37.6 Mb. In comparison to the original YOLOv7 algorithm, it boasts a 4.9% increase in detection accuracy and a 5.3% improvement in recall rate, with a mere 1.8% increase in model parameters. The YOLOv7-E object detection algorithm presented in this study enables precise inflorescence detection and localization across an entire tree at varying distances, offering robust technical support for differentiated and precise blossom thinning operations by thinning machinery in the future.

## 1 Introduction

An excessive number of blossoms on fruit trees can lead to unnecessary nutrient depletion, insufficient resources for fruit development, resulting in numerous but small-sized fruits with low sugar content and poor quality (Kweon and Sagong, 2021). This increases the probability of larger tree sizes, diminishing the economic benefits of orchards. In some newly established orchards, the lack of effective blossom thinning can disrupt the balance between the tree’s growth and fruiting, adversely affecting the development and formation of tree branches and root systems, thereby extending the orchard’s revenue cycle (Reighard et al., 2015).With the continuous advancement of technological capabilities, there is an increasingly profound understanding of the relationship between the load capacity of branches, the balance between nutritional supply and consumption in tree canopies, and the correlation between the amount of retained blossoms and fruiting rates in fruit trees (Liu et al., 2017). Thinning blossoms not only serves to control fruit quality but can also be employed to estimate optimal yields. The distribution and total number of fruits significantly impact fruit size and other quality parameters, making blossom thinning a key technique in regulating both size and quality (Xia et al., 2022).

Iwanami et al. (2018) proposed a crop load management technique based on determining the optimal number of fruits per inflorescence over a decade. According to their findings, effective blossom thinning during the flowering period of fruit trees, guided by the optimal number of fruits per inflorescence, can maintain total fruit yield while effectively enhancing fruit quality. Iwanami et al. (2019) focused on Fuji apples and, over a two-year period, developed a theoretical model elucidating the relationships among blossom thinning timing, crop load, fruit weight, and flowering. The study revealed that when there are three fruits per square centimeter of branch cross-sectional area, the individual fruit weight is 270 g. However, with a crop load of six fruits per square centimeter of branch cross-sectional area, the individual fruit weight decreases to 180 g. This research underscores the significance of efficient blossom thinning during the flowering period of fruit trees, demonstrating its crucial role in extending the peak fruiting period, ensuring fruit quality, and enhancing overall fruit tree yield.

In practical orchard management, orchard owners aim to maximize economic returns by removing excess pear blossoms during orchard blossom period. Blossom thinning methods include manual, chemical, and mechanical thinning. However, these three methods primarily focus on the sole objective of reducing the number of blossoms. They often overlook the scientific requirements regarding the quantity and spatial distribution of blossoms on pear tree branches (Reighard et al., 2015). This limitation arises because both manual and mechanical thinning rely on human visual observation, making it challenging to accurately assess blossom density (Kon et al., 2013). With technological advancements, orchard owners increasingly seek intelligent blossom thinning operations. This approach allows them to save on labor costs while simultaneously determining fruit yield and quality from the thinning phase. Therefore, there is a need for intelligent blossom thinning machinery capable of precise and rapid thinning (Palacios et al., 2020). However, the prerequisite for intelligent blossom thinning machinery is the real-time and accurate acquisition of information about pear tree inflorescences (Farjon et al., 2020).

In recent years, with the continuous innovation of deep learning networks, the application of computer vision technology in the field of agriculture has become increasingly widespread, and the technological bottlenecks for accurate detection of fruit tree blossoms are diminishing (Xu et al., 2021). Tian et al. (2020) proposed an apple blossom segmentation algorithm based on the U-Net backbone network. They improved the Mask RCNN (Girshick, 2015) head network using the U-Net backbone (He et al., 2017), enhancing the original network’s utilization of image features. The segmentation accuracy for apple blossoms at different stages reached 96.43%, with a recall rate of 95.90%. Wang et al. (2020) introduced a pixel-level apple blossom segmentation algorithm based on a fully convolutional network. The F_1_ score on low-resolution images reached 0.85. However, this type of algorithm is susceptible to lighting conditions and lacks robustness. Zhang et al. (2022) used drones to capture RGB images of fruit trees and matched these images with three-dimensional point cloud information from the trees. This approach enabled the visualization-based estimation of apple tree inflorescence density. While this method offers high accuracy and excellent visualization, the process of fruit tree 3D reconstruction and point cloud processing involves significant computational demands. The fitting of RGB images with point clouds is slow and cannot meet the requirements of real-time detection (Tian et al., 2020).

In recent years, one-stage object detection algorithms, with the YOLO series as a representative example, have undergone continuous iterations and updates. These algorithms are characterized by their fast detection speed, high accuracy, and real-time output of detected object categories and positions, making them better suited for the practical requirements of blossom thinning work (Xia et al., 2022).

Wu et al. (2020) improved the YOLOv4 object detection algorithm by implementing channel reduction. This simplification of the model network maintained the accuracy of apple blossom detection, achieving an average detection accuracy of 97.31% across three apple varieties: Fuji, Red Delicious, and Gala. Li et al. (2022) proposed a method for kiwifruit blossom recognition and localization based on YOLOv5l. The method achieved an average accuracy of 91.60%, with a high matching accuracy of 97.60% for identifying individual blossoms and clusters. Shang et al. (2023) introduced an apple blossom detection method based on the YOLOv5S algorithm. This model utilized Ghost modules and ShuffleNetv2 modules to replace the Conv modules in the original network’s Neck section and backbone network. The detection accuracy for apple blossoms under various weather conditions was 88.40%, with a recall rate of 86.10%. The mean average precision was 91.80%. However, the input images mainly consisted of close-up shots and did not satisfy the actual requirements for blossom thinning at a distance. Xia et al. (2023) proposed a whole-tree object detection algorithm by incorporating the Spatial Temporal Pyramid Attention Feature Pyramid Network (He et al., 2015) into the MTYOLOX backbone network. This enhancement increased the network’s focus on small target blossoms, resulting in a precision and recall rate of 83.4% and 93.3%, respectively. Additionally, it facilitated tree-level blossom density mapping. However, this method targeted early-stage apple tree blossoms, which exhibit uniform features and no leaf occlusion. Further research is needed to address scenarios where blossoms overlap and branches or leaves obstruct the view in practical blossom thinning operations.

In summary, both two-stage and one-stage object detection algorithms have made significant advancements in the field of fruit blossom detection. However, they also face certain challenges. Blossom detection typically occurs during the early or late stages of inflorescence, and there is limited research on reducing false negatives and distinguishing between blossoms and buds, especially in scenarios where blossoms heavily overlap during actual blossom thinning operations (Wu et al., 2020). Most detection scenarios involve close-up shots of individual blossoms, and long-range detection is limited by the convolutional layers’ ability to capture only local relationships. Although reducing the number of channels has been employed to enhance inflorescence discrimination, it often neglects the extraction of precise positional information of inflorescences.

In light of the current research status on inflorescence recognition, and to further advance the development of intelligent blossom thinning, this paper focuses on pear tree inflorescences as the detection target. Building upon the YOLOv7 neural network model, we propose a YOLOv7-E object detection algorithm to address the following issues:

(1) Currently, most applications of object detection algorithms involve close-up shots of blossoms, with limited research on long-distance tree-level inflorescence detection. This does not align with the normal working distance requirements for blossom thinning equipment.
(2) Existing studies often concentrate on early or late stages of inflorescence development. However, given the rapid changes during the flowering period of fruit trees, it is impractical for blossom thinning operations to be completed entirely within a specific time frame. In practical blossom thinning work, the object detection algorithm needs to adapt to the varying characteristics of blossoms at different stages, supporting continuous operation of intelligent blossom thinning equipment throughout the entire period.
(3) In the context of long-distance tree-level inflorescence recognition, as the distance increases, discernible features of blossoms in input images become smaller, leading to increased overlap between flowers and greater influence from leaf occlusion. Addressing how to reduce the probability of missed detections and false positives in long-distance detection scenarios is crucial.

The work outlined in this paper is structured as follows:

Chapter 2 begins by introducing the sources and classification of the datasets used in the experiments. It then proceeds to elucidate the methods employed and the evaluation metrics for enhancing the original YOLOv7 object detection algorithm.
Chapter 3 provides an in-depth explanation of the experimental details and carries out experiments to address the established research objectives.
Chapter 4 validates the effectiveness of the proposed methods and conducts comparative evaluations with similar detection algorithms under the same conditions. This validation aims to achieve precise blossom detection on entire trees at long distances. Finally, based on the experimental result, the paper concludes its research findings and presents future prospects.

## 2 Data and methods

### 2.1 Data

The dataset in this study consists of pear tree inflorescence images, collected from the pear orchard at the Fruit Tree Institute of Jiangsu Academy of Agricultural Sciences. The collection period spans from March 2, 2023, to March 27, 2023, covering an entire pear tree flowering season. Data was gathered between 14:00 and 16:00. The collection device used was a Huawei AL10 smartphone with a resolution of 3000×4000 pixels and a focal length of 26 mm.

The inflorescence images in this research are categorized into A, B, and C classes. The specific experimental data is outlined in **Table 1**. To avoid potential overfitting due to insufficient dataset size, data augmentation techniques were applied to the A, B, and C datasets using the OpenCV library. Geometric transformations such as rotation, translation, jittering, and splicing, as well as pixel changes such as Gaussian noise, HSV contrast adjustment, and histogram equalization, were employed. Each augmented image was expanded fivefold, resulting in a final dataset comprising 3,390 images. The training, validation, and test sets were proportionally composed of A, B, and C class images in a 3:4:3 ratio. LabelImg software was used for inflorescence image preprocessing, and the flower category classification included two classes: flowers and flower buds.

**Table 1:** Details of the captured images.

Name	Influencing factors	Specific categories	Number
A	Time	early stage mid-stage middle stage	165 280 335
B	Distance	close-up (10-30 cm) branch (40-60 cm) panoramic (80-120 cm)	130 300 730
C	Weather	sunny cloudy overcast	420 135 330

**Figure 1:**
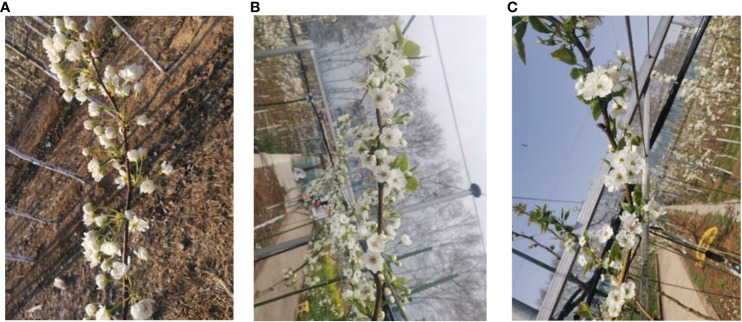
Inflorescence at different stages. **(A)** Early stage **(B)** Mid-stage **(C)** Late stage.

The inflorescence characteristics of pear trees vary significantly at different stages. In the early stages, there is minimal overlap among blossoms, and the inflorescence features primarily consist of flower buds with almost no leaf occlusion. In the middle stages, blossoms coexist with flower buds, and leaf occlusion is generally moderate. In the later stages, the inflorescence features are predominantly blossoms, with a high degree of overlap and mutual occlusion among them. To ensure that the proposed model accommodates inflorescence detection at different stages, the A-class dataset, as depicted in **Figures 1A–C**, includes inflorescence images from the early, middle, and late stages.

**Figure 2:**
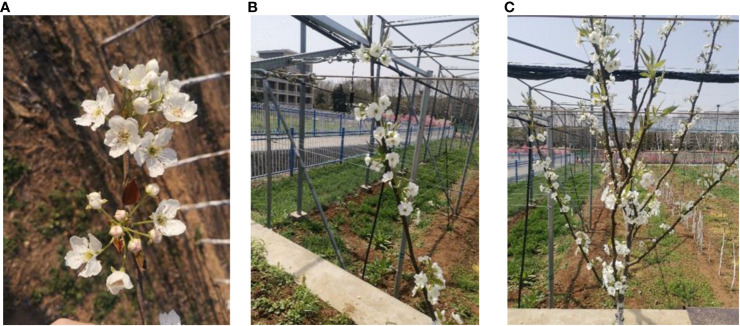
Inflorescence at different distances. **(A)** Close-up **(B)** Branches **(C)** Panoramic.

With increasing distance, the pixel values of inflorescences in input images decrease, and the difficulty of extracting effective image features increases due to the combined effects of leaf occlusion and blossom overlap. To enable the proposed model to adapt to inflorescence detection at different distances, the B-class dataset, as illustrated in **Figures 2A–C**, includes inflorescence images taken at close-up (10-30 cm), intermediate (40-60 cm), and panoramic (80-120 cm) distances.

**Figure 3:**
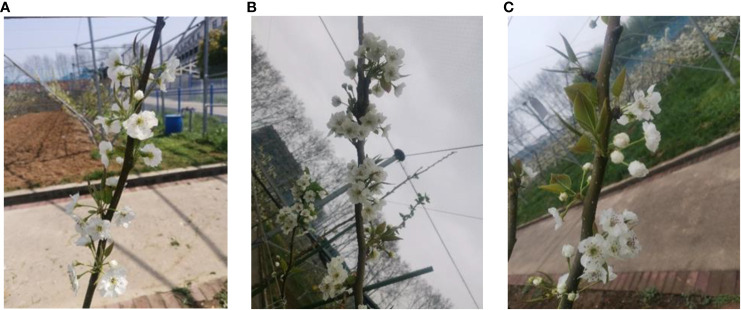
Inflorescence in different weather conditions. **(A)** Sunny **(B)** Overcast **(C)** Rainy.

Variations in lighting conditions are a crucial factor to consider in target recognition. Blossom thinning operations often occur on sunny days; however, the weather during the flowering period of fruit trees is variable. To ensure that the proposed model can achieve inflorescence detection under challenging lighting conditions, the C-class dataset, as depicted in **Figures 3A–C**, comprises inflorescence images taken on sunny, overcast, and rainy days.

### 2.2 Methods

#### 2.2.1 YOLOv7 network

**Figure 4:**
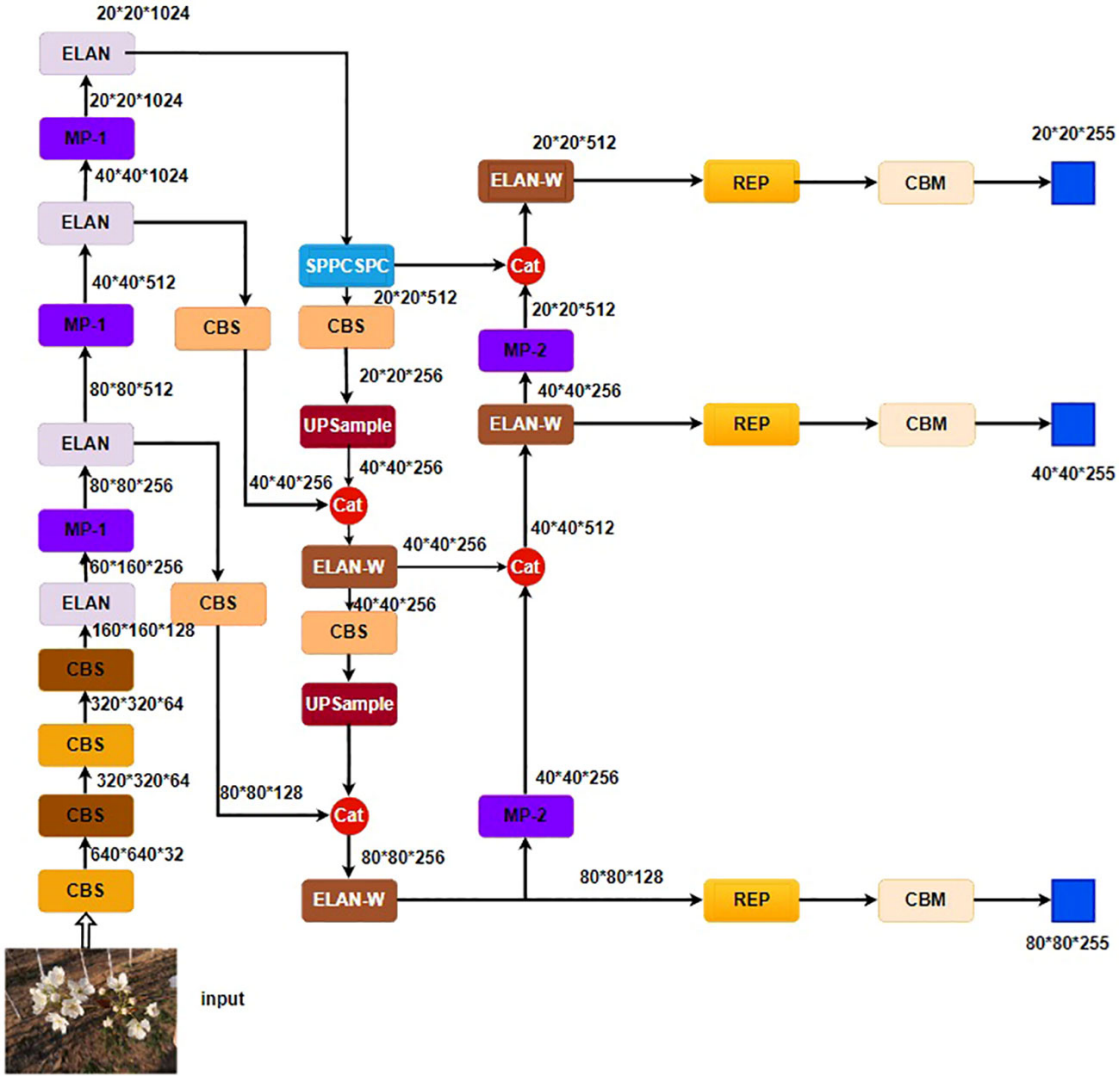
YOLOv7 network architecture.

YOLOv7 (Xia et al., 2023) stands as the current leading object detection algorithm, offering optimal speed ranging from 5FPS to 160FPS while maintaining accuracy. Its network architecture, as illustrated in **Figure 4**, comprises the input, backbone, and head components. Image inputs undergo preprocessing before being fed into the Backbone network for feature extraction. The input to the head layer consists of three feature maps of different sizes. These maps are processed using Rep VGG blocks and convolution layers for tasks such as image classification, foreground-background classification, and bounding box refinement, ultimately yielding the detection results (Chen et al., 2017).

What sets YOLOv7 apart from previous detectors in the YOLO series is its foundation on the Rep VGG structure (Ding et al., 2021). It introduces a novel reparameterization convolution module, which accelerates network inference performance without sacrificing accuracy. Additionally, a coarse-to-fine label assignment method is proposed, wherein initial training utilizes the prediction results from a guidance head, increasing the number of positive samples to expedite training efficiency. The optimal results are subsequently selected based on precision. The ELAN-W module extends the feature dimension of channel and computational modules using three distinct convolution combinations. It merges different features through shuffle and merge cardinality methods, gradually enhancing the network’s capacity for diverse feature learning without compromising the existing gradient pathways (Zhao et al., 2023).The unique label assignment strategy, efficient aggregation network, and reparameterization methods of YOLOv7 are well-suited to address the detection scenarios posed by pear tree inflorescence images, which involve a high number of small-sized blossoms with similar features.

#### 2.2.2 Multi-scale attention module

**Figure 5:**
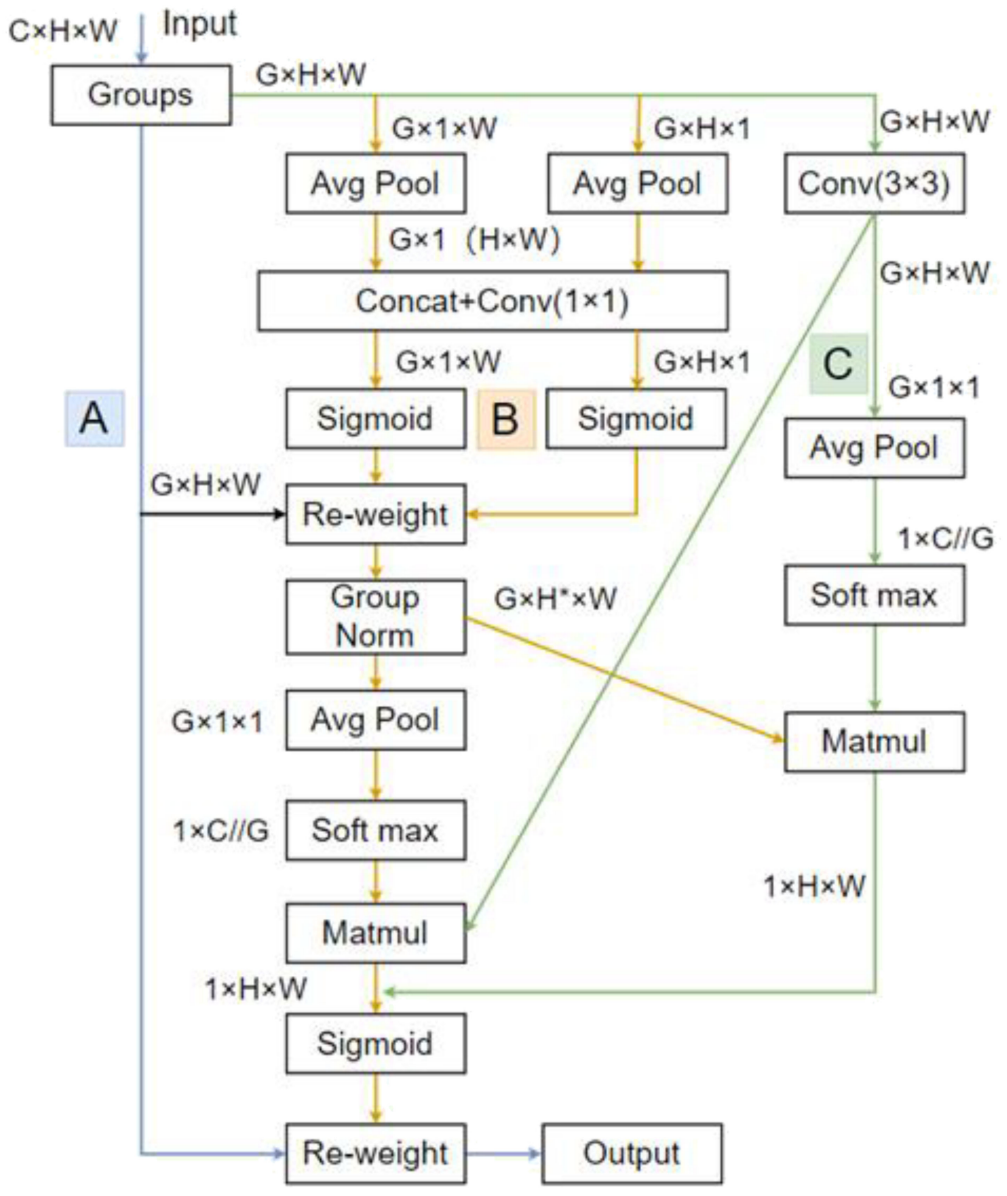
EMA architecture.

While ensuring the accuracy of flower recognition, it is essential to detect the precise spatial information of the blossoms, which is a necessary condition for ensuring the precise operation of the thinning equipment. Detecting inflorescences on an entire tree poses a significant challenge. As the distance increases, the size of the target blossoms in the image becomes progressively smaller. This diminishes the contrast, making it difficult to label and identify inflorescences. To enhance the YOLOv7 algorithm’s capability for detecting small, distant targets, a novel Efficient Multi-Scale Attention (EMA) (Ouyang et al., 2023) mechanism is introduced, as shown in the structural diagram in **Figure 5**. EMA is an efficient multi-scale attention mechanism based on Coordinate Attention (CA). It employs a parallel strategy to divide the original input feature map of size *CHW* into G (G≤C) sub-features. Three path ways, A, B, and C, are utilized to extract feature information weights from different channels. The A branch represents the original input feature map, while the B branch consists of feature maps with sizes *G×1×W* and *G×H×1*. After applying global average pooling, these branches retain feature information in the vertical and horizontal directions. Subsequently, one *1x1* convolution is applied to share similar features, resulting in two *1D* feature encoding vectors. These vectors are then processed with a Sigmoid function to adjust the encoding weights for precise spatial information. The feature map in the C branch undergoes one *3×3* convolution to obtain finer-grained local channel features without significantly increasing computational complexity. To further collect spatial information at multiple scales without reducing channel dimensions or increasing computational load, the channel attention maps from the C branch, which have not been subjected to normalization probability processing, are multiplied by the attention maps from the B branch. This multiplication is performed after applying Group Normalization to the B branch’s channel attention maps (Simonyan et al., 2014).

In a nutshell, EMA achieves the linkage between spatial positions and channels through a parallel structure. It encodes global positional information for both the B and C branches by utilizing two-dimensional global average pooling. This encoding is established through simple multiplicative operations, creating a set of spatial attention weight values. This approach maximizes the capture of pixel-level relationships across the feature map while retaining precise positional information. Moreover, the Group Normalization (Wu and He, 2018) applied to the B branch is unaffected by batch size variations, rendering it highly advantageous in scenarios involving long-distance small targets with high similarity.

It should be noted that the EMA attention mechanism performs exponential weighted averaging on historical attention weights. This process, particularly in scenarios involving long sequences and large-scale models, can lead to an increase in computational complexity. Furthermore, during the application, continuous adjustments to the decay factor are necessary, introducing added difficulty to the model tuning process.

#### 2.2.3 SPPCSPCS module

As neural network designs continue to evolve, enhancing algorithm performance using methods like NAS has become increasingly challenging (Gao et al., 2018). In YOLOv7, we have taken a foundational approach to optimize network layers, aiming to improve the accuracy of the original network without significantly increasing computational costs. In practical applications, variations in the distance between image capture devices and pear trees result in differences in the sizes of pear inflorescences within the input network. Inflorescences are less noticeable as the distance increases, and they appear larger when the distance is shorter. Furthermore, different image capture devices have varying image resolutions. To enhance the algorithm’s ability to handle a broader range of scale features and improve compatibility with edge devices, solely adding attention mechanisms is insufficient to achieve optimal results.

**Figure 6:**
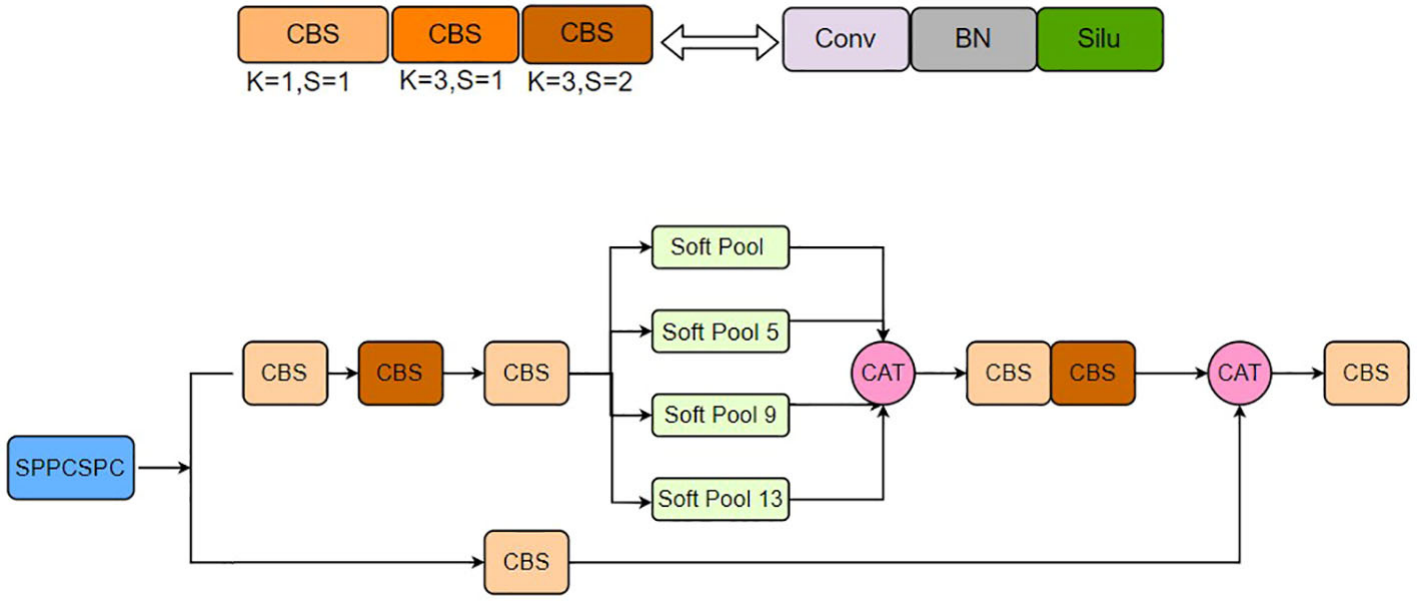
SPPCSPCS architecture.

Therefore, we proposed an improved SPPCSPCS module based on the original SPPCSPC module within the network, as illustrated in **Figure 6**. Initially, input features are split into two branches. The first branch undergoes conventional processing with a 1×1 convolution having a stride of one. The second branch first passes through four different pooling layers for multi-scale feature fusion, followed by a 1×1 convolution with a stride of one and a 3×3 convolution with a stride of two. Finally, the outputs of the two branches are merged, resulting in a minimal increase in computational load while significantly improving the model’s accuracy and compatibility with images of varying resolutions.

Based on the characteristics of actual inflorescence images, the original network architecture’s SPPCSPC module utilizes max-pooling. However, in inflorescence images, neighboring and similar features are abundant, and applying max-pooling tends to retain only the most prominent features, making it prone to missing the detection of identical objects. Soft-pooling (Stergiou et al., 2021) employs a weighted approach based on soft-max to retain the original attributes of the input while enhancing features. Its computation is defined as in Equations 1 and 2.


(1) $$W_{\text{i}}=\frac{e^{a_{i}}}{\sum_{j\in R}e^{a_{j}}}$$

Where, *R* represents the selected local region, *a* denotes a feature value, and *W_i_* represents the weight of the feature value.


(2) $$\tilde{a}=\sum_{i\in R}W_{i}a_{i}$$

Where, $\tilde{a}$ represents the summation of the product of relevant feature values and their corresponding weights.

The Soft-pooling method begins by calculating the weights of the corresponding feature values within the selected regions through an exponential computation. Subsequently, it multiplies each feature value by its respective weight and performs a weighted sum. This approach allows for a comprehensive consideration of feature values from all regions, categorizing important features based on weight magnitudes. In contrast to the direct selection of the maximum value in the former approach, Soft-pooling retains more information. Additionally, Soft-pooling is differentiable, which means it can provide minimal gradient values during the backpropagation process, making it more conducive to model training.

#### 2.2.4 Soft-NMS

In computer vision technology, generating corresponding bounding boxes for target categories has always been a fundamental challenge, especially in tasks involving densely occluded object detection. The process of filtering candidate bounding boxes is directly linked to the algorithm’s detection accuracy, as cited in reference (Tychsen-Smith and Petersson, 2018). During our use of the YOLOv7 network for pre-training, we observed that the non-maximum suppression (NMS) method used by YOLOv7 resulted in numerous missed detections and false alarms in scenarios where pear blossoms are densely clustered and occluding each other (Hosang et al., 2017).

**Figure 7:**
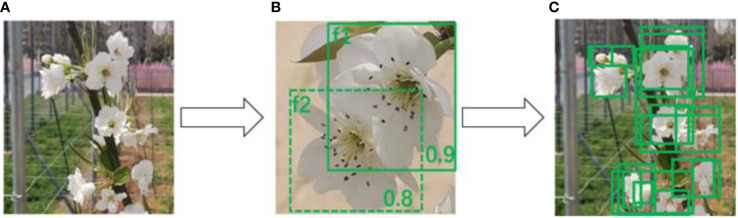
NMS detection performance.

As illustrated in **Figures 7A, B**, the scores for two pear blossoms, *f*_1_ and *f*_2_, are 0.9 and 0.8, respectively. According to the NMS strategy, despite *f*_2_ having a high score of 0.8, it would still be deleted due to the excessive overlap with *f*_1_ and leading to a missed detection. If we simply raise the NMS threshold, it could easily result in false alarms as depicted in **Figure 7C**.

**Figure 8:**
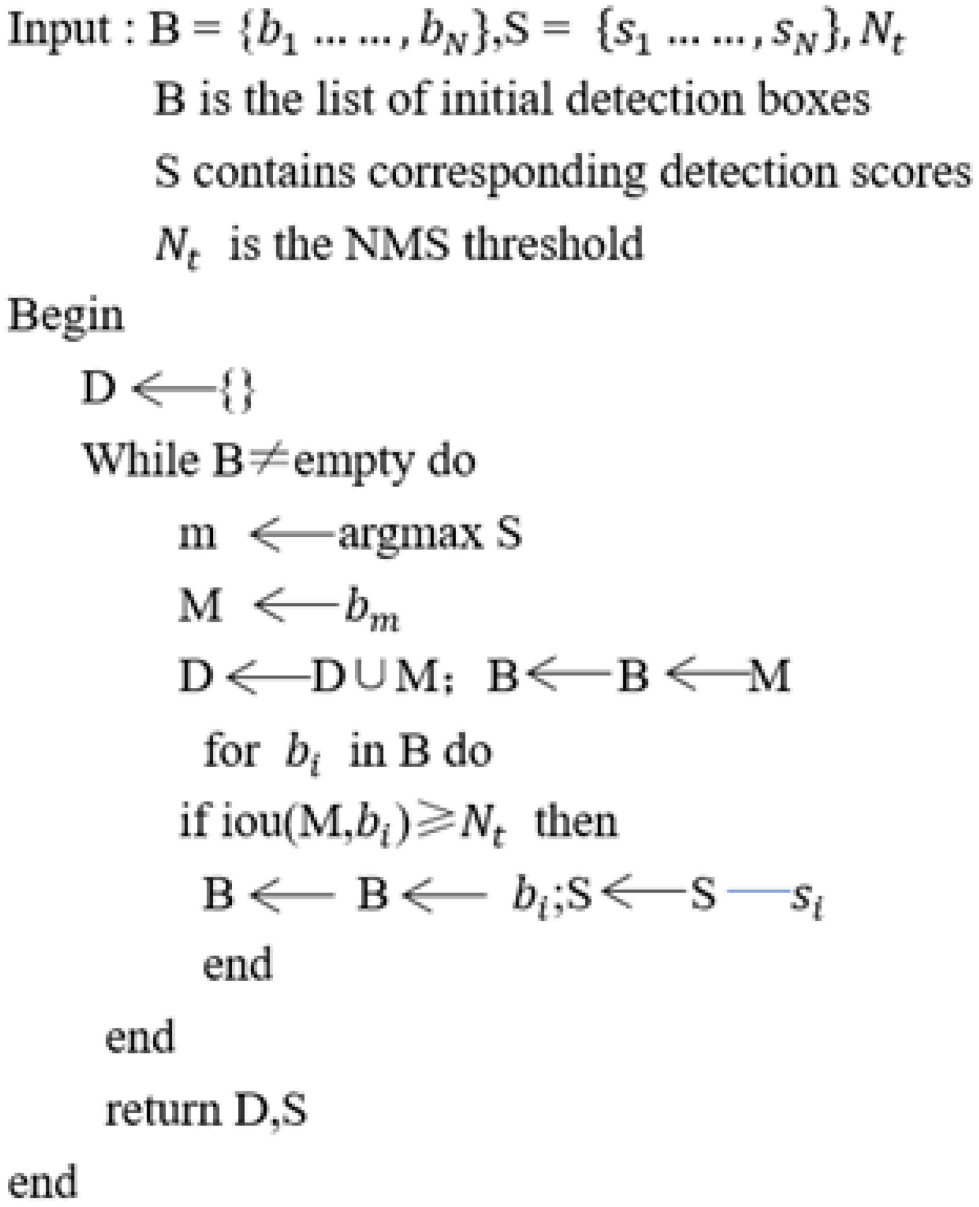
The concept of the Soft-NMS algorithm.


In response to the aforementioned scenario, the Soft Non-Maximum Suppression method is introduced (Bodla et al., 2017). The algorithm conceptual pseudo code is illustrated in **Figure 8**. In this method, *B* represents the collection of scores for all candidate bounding boxes. After obtaining the highest score *M*, it is extracted from the *B* collection and added to the final detection box set *D*. Simultaneously, for candidate bounding boxes in the *B* set that have an overlap with *M* greater than the threshold *N_t_*, a lower score *S_i_* is assigned. What sets Soft-NMS apart from traditional NMS is its approach to candidate bounding boxes with the same overlap values. Instead of directly removing them, Soft-NMS applies a decay function. In simpler terms, if a candidate bounding box significantly overlaps with the bounding box with the highest score *M*, it is assigned a lower score rather than being eliminated. This approach helps prevent missed detections. The calculation of the decay function *Si* is defined as shown in Equation 3.

(3) $$S_{i}=S_{i}e^{-\frac{IoU{\left(M,b_{i}\right)}^{2}}{\sigma }}$$

Where, *S_i_* is equivalent to the confidence score *b_i_* of the prior box, *M* represents the prior box with the highest confidence score in the confidence branch, and *σ* is a hyper parameter.

It is important to note that while Soft-NMS does not impact model size and is plug-and-play, it exhibits sensitivity to the sizes and shapes of bounding boxes. In scenarios where there is a significant disparity in object sizes, Soft-NMS may not perform as well as traditional NMS. Therefore, if applied to detect diverse floral arrangements, adjustments to the decay function parameters are necessary.

### 2.3 Evaluation metrics

This study employs four metrics, precision (P), recall (R), mean average precision (mAP), and F1 score, to evaluate the accuracy of the proposed pear blossom detection model. Model efficiency is assessed based on model parameters, frames per second (FPS), and floating-point operations per second (FLOPS). The formulas for these metrics are provided in Equations 4–7.


(4) $$P=\frac{TP}{TP+FP}\times 100\%$$

(5) $$R=\frac{TP}{TP+FN}\times 100\%$$

(6) $$mAP=\frac{\sum AP}{N_{i}}$$

(7) $$F_{1}=\frac{2\left(P\times R\right)}{P+R}$$

Where, true positives (TP), false positives (FP), and false negatives (FN) represent the number of detection boxes correctly predicting positive samples, the number of detection boxes incorrectly predicting positive samples when they are negative, and the number of actual positive samples incorrectly predicted as negative, respectively. The mean average precision (mAP) measures the model performance across each class. *N_i_* represents the total number of classes the model can detect. In this study, there are two detection classes: buds and blossoms, thus *N_i_* =2.

## 3 Results and discussions

### 3.1 Experimental details

All experiments in this study were conducted on a desktop server equipped with an Intel Core i5-13600 (3.49 GHz) CPU, an NVIDIA Tesla A100 (80 GB) GPU, and 125 GB of RAM. The software environment included Windows 10 Professional, CUDA 11.8, Python 3.8, and PyTorch 1.13 deep learning frame work. During the training phase, the initial weights were initialized using YOLOv7 weights from the CoCo dataset. Our training strategy involved the use of weighted image strategy and multi-scale training methods to address the class imbalance issue, enhance model robustness, and cache images in memory for faster training. The training parameters for the model are summarized in **Table 2**.

**Table 2:** Model training parameters.

Training parameters	Values
Epochs	300
Batch-size	16
Img-size	1280×1280
Initial learning rate	0.001
Warmup epochs	5
Lrf	0.1
Weight_decay	0.0005

### 3.2 Results

#### 3.2.1 Visual detection results of YOLOv7-E

**Figure 9:**
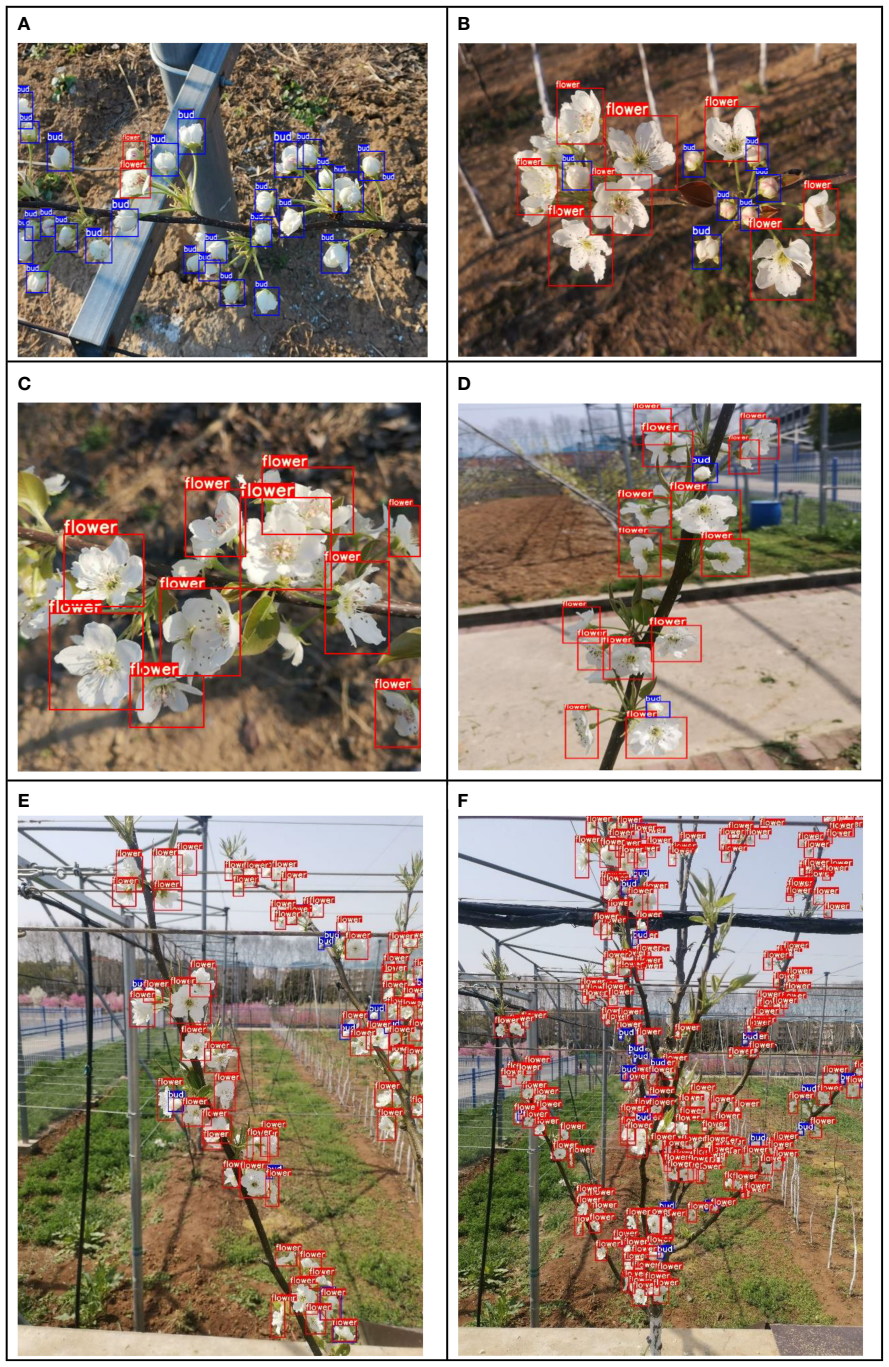
Detection results in different scenarios. **(A)** Early stage **(B)** Mid-stage **(C)** Late stage **(D)** Close-up **(E)** Branches **(F)** Whole tree.

To validate the detection performance of the proposed model across different stages of pear tree inflorescences, testing was conducted using images from dataset A representing various inflorescence stages (with no significant changes in distance and weather conditions). **Figures 9A–C** depict the detection results for the early, middle, and late stages of inflorescences, respectively. According to the evaluation, the YOLOv7-E model achieves an average precision of 92.27%, 91.24%, and 90.06% for the early, middle, and late stages of pear inflorescences, with a variance of 0.8. The results demonstrate that the proposed model can accurately identify pear inflorescences across different stages and possesses the capability for inflorescence detection throughout the entire flowering period.

To validate the inflorescence detection performance of the proposed model at different distances, testing was conducted using inflorescence images from the B-class dataset captured at various distances. **Figures 9D–F** present the detection results for late-stage pear inflorescences at distances of 30 、60 and 120 centimeters, respectively. Upon evaluation, the average precision of detection was determined to be 91.51%, 90.41%, and 89.43% for the respective distances, with a variance of 0.7. The results indicate that the YOLOv7-E model maintains consistent precision under varying distances, aligning with the practical requirements of blossom thinning operations.

**Figure 10:**
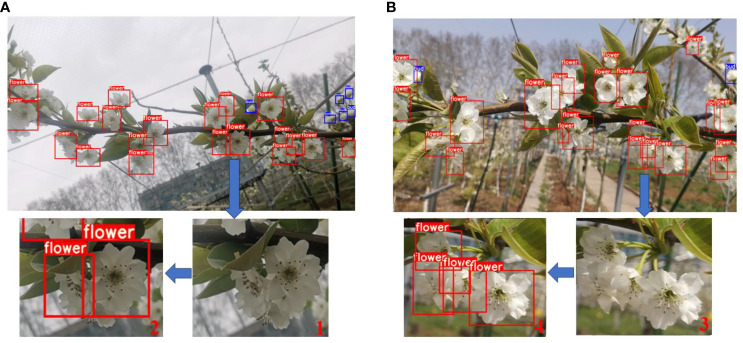
Detection results under different lighting conditions. **(A)** Overcast **(B)** Sunny.

As it is well known, blossom thinning operations predominantly occur on sunny days; however, the weather during the flowering period is subject to variability. To validate the inflorescence detection performance of the improved model under different weather conditions, testing was conducted using inflorescence images from the C-class dataset captured under diverse weather conditions. **Figures 10A, B** depict the detection results for late-stage pear inflorescences (distance: 30-40 cm) on overcast and sunny days, respectively. Upon evaluation, the average precision of detection under overcast and sunny conditions was determined to be 88.14% and 91.28%, respectively. As illustrated in **Figure 10** (1, 2, 3, 4), whether on overcast or sunny days, the YOLOv7-E model demonstrates the capability to correctly identify occluded blossoms even in situations of high blossom overlap. The results indicate that the proposed model achieves precise inflorescence detection under varying weather conditions.

### 3.3 Discussion

#### 3.3.1 Ablation experiment

In order to assess the effectiveness of the interactions among the various modules in the proposed YOLOv7-E model, this study conducted ablation experiments using the same training and test datasets. The performance metrics for each model are presented in **Table 3**. In **Table 2**, YOLOv7 represents the original YOLOv7 model, YOLOv7-EMAindicates the model with the efficient attention mechanism introduced, YOLOv7-SPPCSPCS represents the model with the improved SPPCSPCS module, and YOLOv7-Soft NMS is the model using the Soft Non-Maximum Suppression method.

**Table 3:** Performance parameter comparison between improved YOLOv7 and original YOLOv7.

Algorithm	mAP/%	Recall%	F1%	GFLOPs	Average speed/(ms)	Model size/M
YOLOv7	86.5	84.5	85.4	104.8	74.2	36.9
YOLOv7-EMA	90.7	90.4	90.5	107.3	76.8	37.4
YOLOv7-SPPCSPCS	88.2	86.5	87.3	105.2	74.5	37.1
YOLOv7-Soft NMS	89.6	88.9	89.3	104.5	76.1	36.9
YOLOv7-E	91.4	89.8	90.6	108.7	80.9	37.6

According to **Table 3**, it is evident that the YOLOv7-EMA and YOLOv7-SPPCSPCS models, in comparison to the original YOLOv7 model, exhibit improvements in mAP by 4.2% and 1.7%, respectively. Furthermore, they demonstrate an increase in recall rates by 5.9% and 2%. The model size experiences slight increments of 1% and 0.5%, while the GFLOP increases by 2.3% and 0.3%. However, it’s important to note that the detection speed is slower by 2.6 ms and 0.3 ms for these models. In the case of YOLOv7-Soft NMS, there is a noticeable enhancement in mAP and recall rates by 3.1% and 4.4%, respectively, when compared to the original network model. However, this improvement leads to a slight reduction in detection speed by 1.9 ms. The model size remains unchanged. This indicates that Soft Non-Maximum Suppression, without impacting the original network structure and model size, can significantly improve the original network’s detection accuracy in scenarios with high overlap. This is achieved through the effective allocation of a decay function strategy, which reduces both missed detections and false positives.

For YOLOv7-E, there is a notable increase in mAP and recall rates by 4.9% and 5.3%, respectively, when compared to the original network. However, this improvement comes at the cost of a 3.7% increase in GFLOP and a 1.8% growth in model size. The average detection speed is slightly slower by 6.7 ms. The results suggest that the EMA, based on parallel strategies, effectively captures finer-grained local channel features without significantly increasing computational demands. It does so through the construction of a set of spatial attention weight values, thereby enhancing the capture of pixel-level relationships for small targets on the feature map in long-distance detection scenarios. Additionally, the Soft-pooling-enhanced SPPCSPCS module, as training progresses during backpropagation, accelerates the update of target weight values and retains a greater amount of regional feature information. From the experimental results, it can be concluded that the proposed YOLOv7-E model, with only a minimal increase in model size, effectively enhances the YOLOv7 network model’s accuracy in detecting highly overlapping inflorescences at long distances while maintaining the overall model structure.

#### 3.3.2 Performance comparison of different models

**Figure 11:**
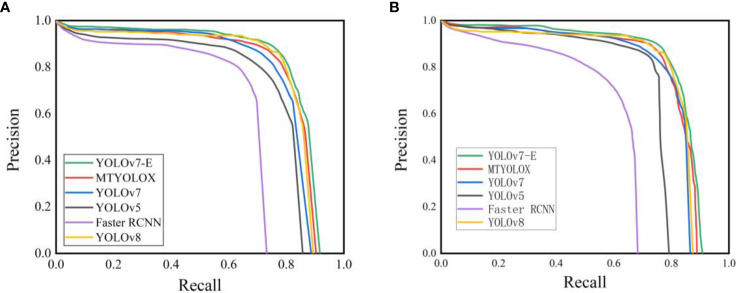
P-R curve. **(A)** P-R curve at 30-60 cm. **(B)** P-R curve at 80-120 cm.

To further validate the effectiveness of the proposed YOLOv7-E network model, this study conducted a comparative analysis with four other object detection algorithms, namely, MTYOLOX、YOLOv7、YOLOv5、Faster R-CNN and YOLOv8. All models were trained using the same training dataset, and the training process consisted of 300 epochs. Given the primary focus of this study on enhancing the original network model’s ability to detect overlapping inflorescences at long distances, testing was performed without the use of a separate test dataset. **Figure 11** displays the Precision-Recall (P-R) curves obtained with different test datasets. In **Figure 11A** represents a test dataset equally divided into three classes (ABC) in a 3/3/4 ratio, while (b) depicts a test dataset with classes (ABC) divided in a 2/6/2 ratio.

From **Figures 11A, B**, it is evident that YOLOv7-E exhibits excellent P-R curves in both close-range and long-range scenarios. This observation suggests that the proposed method can effectively meet the practical requirements of sparse flower operation, enabling accurate detection of pear inflorescences at distances ranging from 80 to 120 centimeter.

As shown in **Table 4**, the improved YOLOv7-E model in this study demonstrates a 4.9% increase in Mean Average Precision (MAP) and a 5.3% increase in recall compared to the original model. While enhancing accuracy, the model also reduces the false-negative rate. Regarding model parameters and inference speed, YOLOv7-E exhibits an increase of 0.7 M in parameter count and 6.7 ms in inference speed compared to the original model. Without compromising detection speed, the slight increase in model parameters results in a noticeable improvement in model inference accuracy, making it an acceptable trade-off.

**Table 4:** Performance comparison of different object detection algorithms.

Algorithm	MAP%	Recall%	F1%	GFLOP	Speed/ms	Model size/M
YOLOv7	86.5	84.5	85.4	104.8	74.2	36.9
MTYOLOX	90.2	92.1	91.1	111.3	89.8	73.2
YOLOv5	80.8	81.2	80.9	89	84.2	46.8
Faster RCNN	71.3	73.4	72.3	206	108.3	106
YOLOv8	87.8	86.2	86.9	145.2	92.5	43.7
YOLOv7-E	91.4	89.8	90.6	108.7	80.9	37.6

The MAP values of the YOLOv7-E model are respectively 1.2%, 10.6%, and 20.1% higher than those of MTYOLOX, YOLOv5, and Faster RCNN object detection algorithms. The recall rate is 8.6% and 16.4% higher than YOLOv5 and Faster-RCNN, and 2.3% lower than MTYOLOX. In terms of detection speed, YOLOv7-E outperforms Faster RCNN, MTYOLOX, YOLOv5, and YOLOv8. Regarding model size, YOLOv7-E is 35.6 M, 68.4 M, 9.2 M, and 6.1 M smaller than MTYOLOX, Faster-RCNN, YOLOv5, and YOLOv8, respectively, making it more convenient for deployment on embedded devices.

It is noteworthy that, in comparison to the latest YOLOv8 object detection algorithm, YOLOv7 exhibits a 1.3% decrease in Mean Average Precision (MAP) and a 1.7% decrease in recall. However, in this study, YOLOv7-E surpasses YOLOv8 in both average precision and recall by 3.6%. This suggests that the Exponential Moving Average (EMA) attention mechanism, through the aggregation of spatial attention weight values, effectively preserves the pixel-level relationships of each inflorescence in the input images. Moreover, by employing a unique decay function, YOLOv7-E captures inflorescence information that was missed in the original model and YOLOv8. Furthermore, YOLOv8 has 5.1M more parameters and 36.5 additional GFLOPS compared to YOLOv7. The results indicate that the approach of YOLOv7-E, which links spatial position and channel information, is more flexible and efficient than YOLOv8. Unlike YOLOv8, which abandons predefined anchor boxes and uses feature maps of different scales to detect targets of various sizes, YOLOv7-E requires fewer computational resources while demonstrating more flexible and efficient performance.

#### 3.3.3 Problem analysis

**Figure 12:**
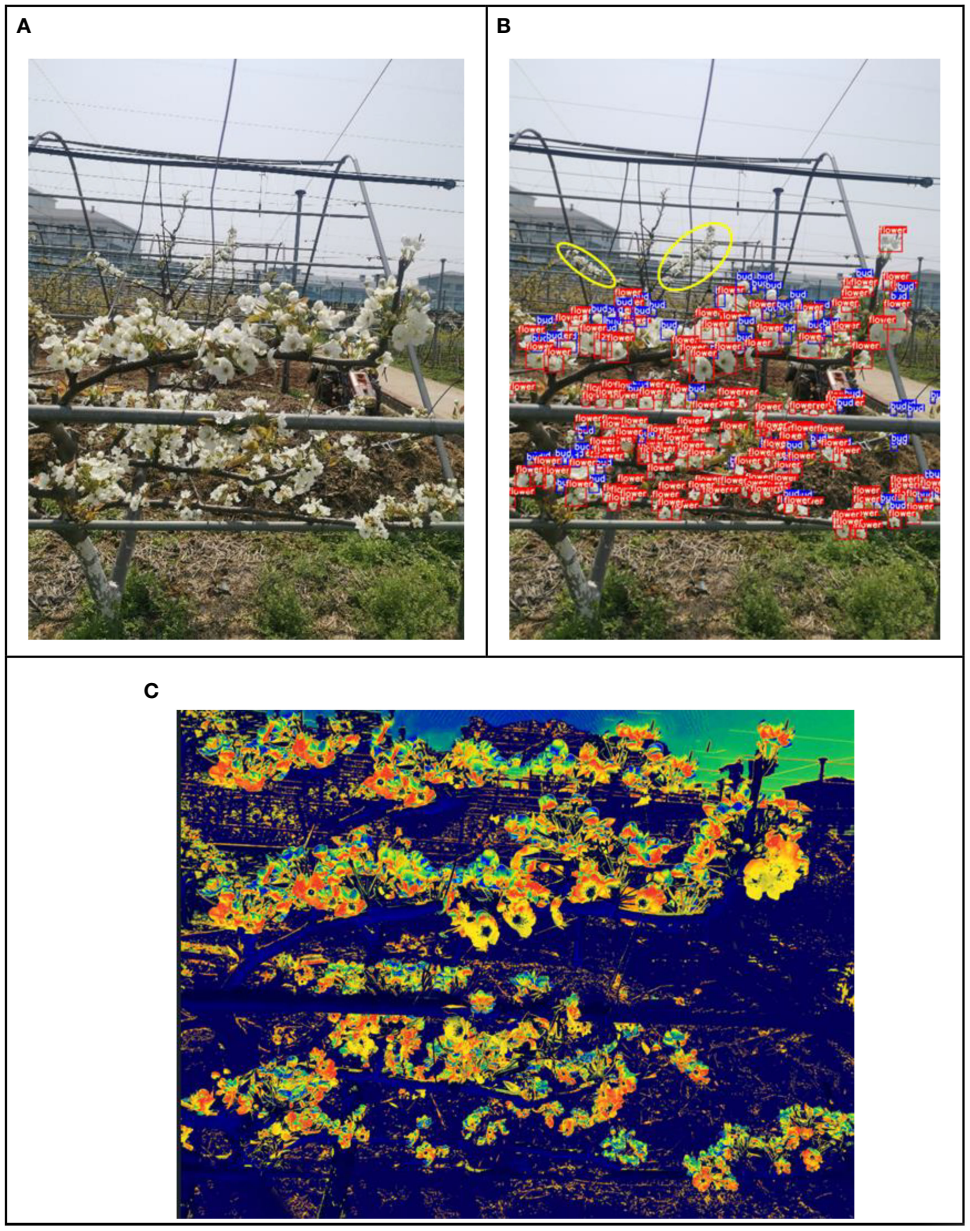
The detection results of the Y-trellis pear tree. **(A)** Y-trellis pear tree **(B)** Detection results **(C)** Class activation map.

To further verify the inflorescence detection capabilities of the improved YOLOv7-E model at operational distances in flower thinning machinery, experiments were conducted on Y-trellis pear trees in the mid-flowering stage at the pear orchard of the Jiangsu Academy of Agricultural Sciences. The detection results are depicted in **Figures 12A–C**. **Figure 12A** shows the real image captured at a distance of 120 cm from the Y-trellis pear tree. **Figure 12B** presents the detection results obtained using YOLOv7-E, which identified 246 blossoms and 73 buds. In **Figure 12C**, the inflorescence detection results were visualized as a heat map using the Grad-CAM (Class Activation Mapping method), with red areas indicating blossoms and blue areas representing buds. The results demonstrate that the EMA attention mechanism and the improved SPPCSPCS module introduced in this study effectively capture the overall inflorescence features at long distances, reduce the focus on irrelevant information, maximize the retention of global image features and positional information. Additionally, the soft-NMS algorithm allows for distinguishing between blossoms and buds, even when they overlap.

As shown in **Figure 12B**, the inflorescence within the yellow circle was not detected. The reasons for this false negative (missed detection) are analyzed as follows.

(1) The pear inflorescence images were captured using a Huawei AL10 smartphone. During photography, the focal point of camera was in the densely populated flower area, causing background blurring within the yellow circle area and resulting in the loss of inflorescence features.
(2) The images were taken on an overcast day, and the inflorescence within the yellow circle was located at a greater distance. Since pear blossoms are white and tend to blend with the color of the sky in the background as the distance increases, it added difficulty to feature extraction, leading to the missed detection.

The edge device utilized for image processing in this experiment is the NVIDIA Jetson AGX Orin (64GB) Developer. With an input resolution of 1024×1024 images, the detection speed reached 40fps, and the detection performance was minimally impacted, marking the achievement of an initial milestone. However, in practical blossom thinning operations, the development kit employed in this study, despite its superior performance, proves cost-prohibitive for integration into blossom thinning equipment. Considering the performance of embedded devices in real-world applications, the proposed YOLOv7-E algorithm faces the following limitations:

(1) The model size is slightly large, occupying excessive memory and limiting the system’s ability to simultaneously run multiple tasks.
(2) The high detection accuracy and lengthy inference time demand significant computational resources, potentially causing performance bottlenecks in embedded systems and compromising real-time responsiveness.
(3) The power consumption during the inference process is substantial, leading to device overheating and requiring high thermal dissipation capabilities in embedded systems.

## 4 Conclusion

With the continuous development of smart agriculture, to facilitate the precision and intelligence of blossom thinning processes, this study, taking into consideration the practical requirements of blossom thinning work, addresses challenges related to high overlap of inflorescences, abundant similar features in pear blossom images, and the difficulty of long-distance detection. In response to these challenges, the study introduces a YOLOv7-E object detection algorithm. The main research findings are as follows.

(1) The YOLOv7-E object detection algorithm proposed in this paper achieves cross-channel feature interaction, maximizing the retention of positional information in inflorescence images captured at long distances. It effectively detects tree-level inflorescences at distances of 80 cm to 120 cm during different stages of pear tree flowering. The average detection precision reaches 91.4%, with a recall rate of 89.8%. The detection speed is measured at 80.9 ms. This ensures that intelligent blossom thinning equipment can operate throughout the entire flowering period of pear trees, adapting to the variable weather conditions, and maintaining effective performance within normal working distances.
(2) The Soft-NMS strategy and the improved SPPCSPCS module introduced in this paper effectively reduce the likelihood of false negatives and false positives when dealing with dense tree-level inflorescences. This approach maximally retains the global feature information of the input inflorescence images, enabling intelligent blossom thinning equipment to more accurately obtain the count of blossoms and buds for each inflorescence on the entire tree. Consequently, based on the varying inflorescence density, the thinning axis rotation speed can be adjusted, providing reliable data support for achieving differentiated and precise blossom thinning.
(3) The YOLOv7-E model proposed in this paper achieves effective detection of tree-level pear inflorescences at long distances in complex scenarios. However, the model exhibits a slightly larger size and demands high computational resources. In the future, there is a need for the model to evolve towards greater lightweight characteristics, enhancing compatibility with edge devices. A potential avenue for improvement is through Model Pruning, where certain threshold-weighted and redundant channel weights are removed. For instance, the introduction of Ghost modules, which partition input channels into two parts and employ shallow convolutions through the Ghost path, can reduce computational load. Alternatively, inspiration can be drawn from the depth-wise separable convolution concept in MobileNetV3, decomposing the standard convolution into depth-wise and point-wise convolutions to maintain performance while reducing model size.

## Data availability statement

The original contributions presented in the study are included in the article/**Supplementary Material**. Further inquiries can be directed to the corresponding authors.

## Author contributions

ZZ: Writing – original draft. XHL: Conceptualization, Funding acquisition, Writing – review & editing, Project administration. KH: Data curation, Formal analysis, Writing – review & editing. YS: Investigation, Software, Writing – review & editing. JZ: Investigation, Supervision, Writing – review & editing. TX: Methodology, Project administration, Writing – review & editing. QY: Software, Validation, Writing – review & editing. YQ: Data curation, Project administration, Writing – review & editing. AH: Funding acquisition, Project administration, Writing – review & editing. XLL: Conceptualization, Funding acquisition, Writing – review & editing.
